# Pathogenic mutations in two families with congenital cataract identified with whole-exome sequencing

**Published:** 2013-02-18

**Authors:** Yukiko Kondo, Hirotomo Saitsu, Toshinobu Miyamoto, Byung Joo Lee, Kiyomi Nishiyama, Mitsuko Nakashima, Yoshinori Tsurusaki, Hiroshi Doi, Noriko Miyake, Jeong Hun Kim, Young Suk Yu, Naomichi Matsumoto

**Affiliations:** 1 Department of Human Genetics, Yokohama City University Graduate School of Medicine, Yokohama 236-0004, Japan; 2 Department of Obstetrics and Gynecology, Asahikawa Medical College, Asahikawa, Japan; 3 Department of Ophthalmology, Seoul National University College of Medicine, Seoul, Korea

## Abstract

**Purpose:**

Congenital cataract is one of the most frequent causes of visual impairment and childhood blindness. Approximately one quarter to one third of congenital cataract cases may have a genetic cause. However, phenotypic variability and genetic heterogeneity hamper correct genetic diagnosis. In this study, we used whole-exome sequencing (WES) to identify pathogenic mutations in two Korean families with congenital cataract.

**Methods:**

Two affected members from each family were pooled and processed for WES. The detected variants were confirmed with direct sequencing.

**Results:**

WES readily identified a *CRYAA* mutation in family A and a *CRYGC* mutation in family B. The c.61C>T (p.R21W) mutation in *CRYAA* has been previously reported in a family with congenital cataract and microcornea. The novel mutation, c.124delT, in *CRYGC* may lead to a premature stop codon (p.C42Afs*60).

**Conclusions:**

This study clearly shows the efficacy of WES for rapid genetic diagnosis of congenital cataract with an unknown cause. WES will be the first choice for clinical services in the near future, providing useful information for genetic counseling and family planning.

## Introduction

Congenital cataract is one of the most frequent causes of visual impairment and childhood blindness worldwide, with an estimated incidence of 2.49 per 10,000 live births by the age of 1 year in the United Kingdom [1]. Congenital cataract is also the leading cause of treatable blindness in childhood. Good outcomes have been reported in children undergoing surgery before 6 weeks of age in bilateral cases [2]. Early diagnosis in the postnatal unit is important for obtaining good visual function.

Many causes have been considered for congenital cataract: intrauterine infection, exposure to drug or radiation in pregnancy, gene defects, chromosomal disorders, metabolic disease, and trauma [3]. Approximately one quarter to one third of congenital cataract cases may have a genetic cause and often follow a Mendelian inheritance pattern, with autosomal dominant traits more common than autosomal recessive and X-linked traits [4,5]. Inter- and intrafamilial phenotypic variability has been reported in cases of inherited congenital cataract [6,7]. It may occur as an isolated eye anomaly, in association with other ocular anomalies, or as part of a systemic disorder. Congenital cataracts are caused by mutations in various types of genes: lens-related crystallin genes (*CRYAA*, *CRYAB*, *CRYBB1*, *CRYBB2*, *CRYBB3*, *CRYBA1*, *CRYBA4*, *CRYGC*, *CRYGD*, and *CRYGS*), membrane protein genes (*GJA3*, *GJA8*, *MIP*, and *LIM2*), cytoskeleton-related genes (*BFSP1* and *BFSP2*), and transcription factor genes (*FOXE3*, *HSF4*, *MAF*, *PITX3*, and *PAX6*) [8]. Weisschuh et al. reported that mutations in crystallin genes occupied 50% of all mutations in known disease-causing genes [9], suggesting that mutations in the crystallin genes are particularly abundant.

Whole-exome sequencing (WES) targeting all the protein-coding genes is powerful and cost-effective for dissecting the genetic basis of diseases [10]. WES is particularly useful for identifying pathogenic mutations for Mendelian disorders for which conventional approaches are difficult (such as when most cases are sporadic).

In this report, we performed WES on two Korean families with congenital cataract inherited in an autosomal dominant fashion. We identified pathogenic mutations in both families and demonstrated the diagnostic utility of WES in congenital cataract.

## Methods

### Clinical report

**Figure 1:**
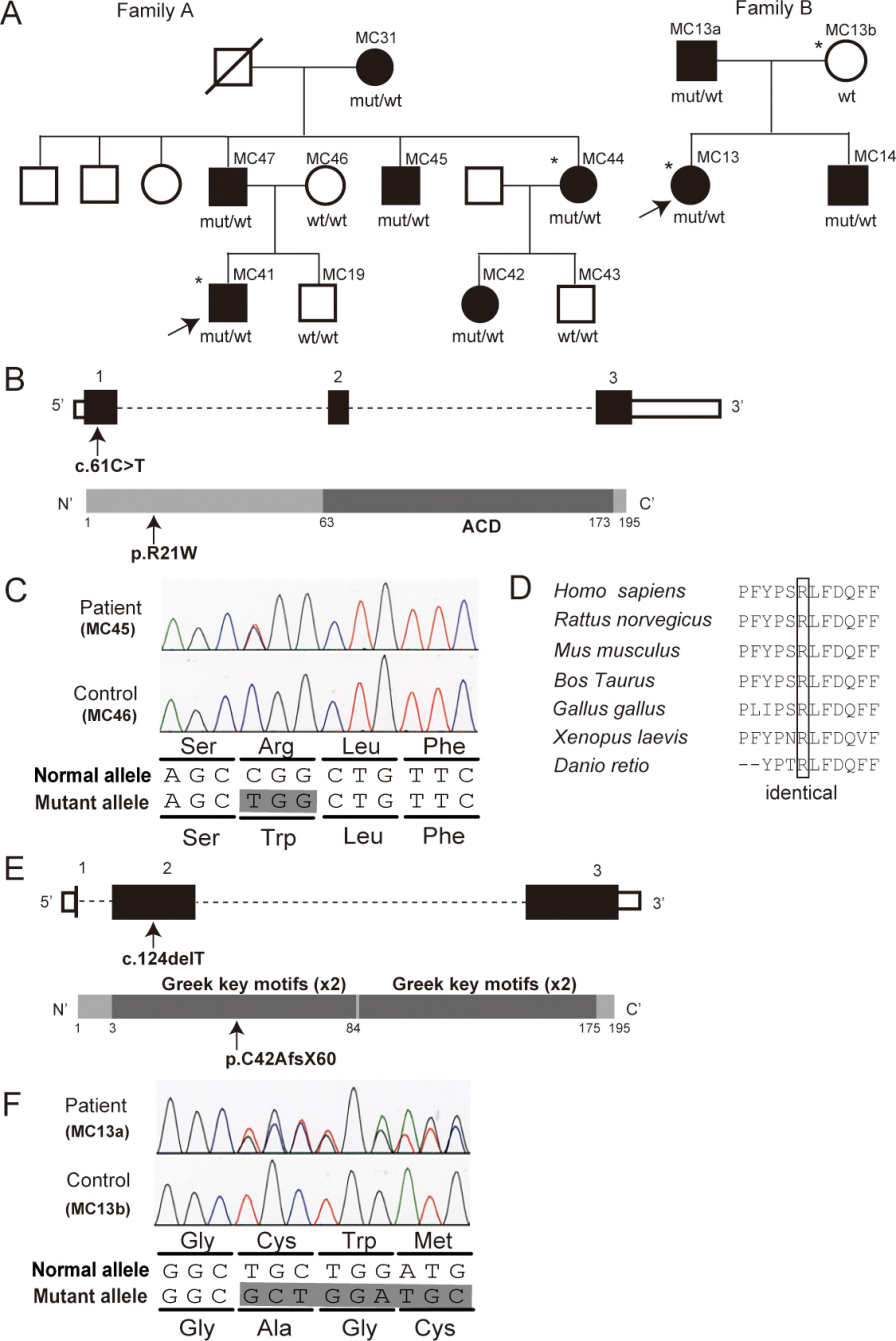
*CRYAA* and *CRYGC* mutations in two Korean families. **A**: Pedigrees of families A and B are indicated. Black and open symbols denote affected and unaffected individuals, respectively. The asterisk shows samples used for whole-exome sequencing. The mutations cosegregate with the phenotype. **B**: Schema of the *CRYAA* gene (top) and the CRYAA protein (bottom) is presented. The untranslated regions and coding region are shown as open and filled rectangles, respectively. The location of the c.61C>T mutation is indicated with an arrow. CRYAA contains an N-terminal region, an α-crystallin domain (ACD, dark gray box), and a C-terminal region. **C**: Electropherograms of the mutation in the affected patient (top) and the unaffected control (bottom) are shown. A single nucleotide change in exon 1 results in an amino acid alteration. **D**: The missense mutation occurred at an evolutionarily conserved amino acid. Homologous sequences were aligned using CLUSTALW. **E**: Schema of the *CRYGC* gene (top) and the CRYGC protein (bottom) is presented. The untranslated regions and the coding region are shown as open and filled rectangles, respectively. The location of the c.124delT mutation is indicated with an arrow. CRYGC contains two domains each composed of two Greek-key motifs (dark gray boxes). **F**: Electropherograms of the CRYGC mutation in the affected patient (top) and in the unaffected control (bottom) are shown. A single nucleotide deletion in exon 2 would cause a frameshift. mut, mutant allele; wt, wild-type allele.

The two Korean families with congenital cataract have been described previously (Figure 1A) [11]. Samples from family A with 6 affected (3 females and 3 males) and 3 unaffected members (1 female and 2 males) and family B with 3 affected (1 female and 2 male) and 1 unaffected (female) members were collected at Seoul National University College of Medicine. In family A, the proband (MC41) was diagnosed with congenital cataract and microphthalmia. Other ocular anomalies were noted, including nystagmus, amblyopia, glaucoma, and esotropia. The cousin of the proband (MC42) showed congenital cataract but no microphthalmia. Nystagmus and amblyopia were also noted. In family B, the older sister (MC13, the proband) and the younger brother (MC14) showed congenital cataract. Other ocular anomalies were found, including nystagmus and amblyopia. Systemic abnormalities, intellectual disability, and developmental malformation were unrecognized, and other possible causes such as trauma, intrauterine infection, exposure to drug or radiation, and metabolic disease were unlikely to be involved in both families.

### Whole-exome sequencing

Blood was collected from antecubital veins of family members and subsequently treated with a Qiagen Blood and Cell Culture DNA Midi Kit (Qiagen) for preparing genomic DNA of leukocytes. Experimental protocols were approved by the Institutional Review Board for Ethical Issues at Yokohama City University School of Medicine and the Committee for Ethical Issues on the Human Genome and Gene Analysis, Seoul National University. Informed consent was obtained from all individuals.

Because the DNA samples were limited, 1.5 μg of DNA from each of two affected members in the respective families were combined, and were processed using a SureSelect Human All Exon 50 Mb Kit (Agilent Technologies, Santa Clara, CA) to generate exome libraries. The libraries were sequenced with one lane per sample of the flow-cell on an Illumina GAIIx (Illumina Inc., San Diego, CA) with 107-bp paired-end reads, according to the manufacturer’s instructions. Image analysis and base calling were performed with Sequence Control Software with Real-Time Analysis (Illumina) and CASAVA software v1.7 (Illumina). Reads were aligned and mapped to the human reference genome sequence (University of California Santa Cruz [UCSC] Genome Browser hg19, National Center for Biotechnology Information [NCBI] genome sequence website build 37) using MAQ [12] and NextGENe software v2.00 with sequence condensation by consolidation (SoftGenetics, State College, PA). Single nucleotide variants (SNVs) were called using MAQ and NextGENe. Small insertions and deletions were detected using NextGENe. Called SNVs were annotated with SeattleSeq Annotation. Candidate variants were confirmed with Sanger sequencing with a 3130xL or 3500xL Genetic Analyzer (Applied Biosystems, Foster City, CA). The Human Gene Mutation Database (HGMD; Biobases, Wolfenbuettel, Germany) was used to check whether the variants had been previously reported. Polymorphism Phenotyping (PolyPhen-2), Sorting Intolerant from Tolerant (SIFT), and MutationTaster were used to evaluate variants in terms of sequence conservation, chemical change, and likelihood of pathogenicity.

## Results

With WES, we attained more than 86% target coverage by ten reads or more (Appendix 1). We adopted a prioritization schema to identify the pathogenic mutation in each pooled sample as follows (Table 1). First, we excluded the variants registered in the Single Nucleotide Polymorphism database (dbSNP132) or the 1000 Genomes project. Then, SNVs commonly detected with MAQ and NextGENe were selected as highly confident variants. In family A, we identified 671 non-synonymous or canonical splice site change SNVs along with 100 small insertions or deletions. We surveyed these for mutations in the 26 known congenital cataract genes and 19 anophthalmia or microphthalmia genes (Appendix 2). We found a heterozygous mutation (c.61C>T [p.R21W]) in exon 1 of *CRYAA* (NM_000394.2), which was confirmed with Sanger sequencing (Figure 1B,C; Table 1). The mutation occurred at an evolutionarily conserved amino acid (Figure 1D), and was previously reported in a family with congenital cataract and microcornea [7]. The mutation completely cosegregated with the cataract phenotype in this family (Figure 1A).

**Table 1:** Sequence variants in the two families found by whole-exome sequencing

	Family A		Family B	
	NextGENe	MAQ	NextGENe	MAQ
Total variant calls	118,801	170,093	130,791	175,155
Unknown SNP variants (dbSNP132, 1000 Genomes project)	28,620	22,038	34,627	21,687
SNVs commonly found by two methods	3,269		2,347	
NS+SP (indels)^a^	671	(100)	454	(135)
Present among 45 candidate genes	1		1	
Confirmed segregation (heterozygous)	1		1	

^a^Small indels were detected only by NextGENe. SNP, single nucleotide polymorphism; SNV, single nucleotide variant; NS, non-synonymous variants; SP, canonical splice site variants; indels, small insertions or deletions.

In family B, we similarly identified 454 non-synonymous or canonical splice site SNVs, and 135 small insertions or deletions (Table 1). We found a novel heterozygous frameshift mutation, c.124delT (p.C42Afs*60) in *CRYGC* (NM_020989.3), and confirmed the presence of the mutation in MC13 but not in MC13b with Sanger sequencing (Figure 1E,F; Table 1). Although we pooled DNA from MC13b and MC13 based on our initial clinical information (Figure 1A), MC13b was actually unaffected (because of an error in information transfer). After the phenotypic information for this family was corrected, the mutation completely cosegregated with the cataract phenotype, as confirmed with Sanger sequencing (Figure 1A). This 1-bp deletion would be expected to result in the insertion of 60 new amino acids after the mutation site with a premature stop codon at position 102 (p.C42Afs*60). This mutation was not found in the National Heart, Lung, and Blood Institute (NHLBI) Exome Sequencing Project (ESP) Exome Variant Server that contains data from more than 5,400 exomes, or among our in-house exome data from 135 individuals.

## Discussion

In this study, a pathogenic mutation in *CRYAA* or *CRYGC*, which encode a crystallin family protein, was identified in each of two Korean families with congenital cataract. Crystallin constitutes the major protein of the vertebrate eye lens and is classified into three main types: α-, β-, and γ-crystallin. *CRYAA*, encoding αA-crystallin, maps to chromosome 21q22.3, and mutations have been reported in autosomal dominant congenital cataract [13]. The αA-crystallin protein consists of an N-terminal region, a conserved α-crystallin domain, and a short C-terminal region. The α-crystallin domain may be involved in aggregating and disaggregating larger protein complexes, whereas the N-terminal and the C-terminal regions are suggested to play a role in oligomerization [7,14,15]. The missense mutation found in family A occurred at an evolutionarily conserved amino acid in the N-terminal region, suggesting that the mutation may impair oligomerization. *CRYGC*, encoding γC-crystallin, plays a crucial role in lens development and the maintenance of lens transparency [16]. The γC-crystallin proteins are tightly folded into two domains, with each domain composed of two exceptionally stable protein structures called Greek-key motifs [17-19]. The relatively loose or partially unfolded structure of mutant γC-crystallin may be susceptible to aggregation and insolubilization, which leads to cataract formation [20]. Ren et al. reported a 5-bp duplication (c.119_123dupGCGGC) within exon 2 of the *CRYGC* gene in patients with autosomal dominant congenital cataract [16]. The c.124delT mutation in family B and the c.119_123dupGCGGC mutation cause truncation within the first domain, and are likely to lead to similar effects.

We pooled DNA from one unaffected case (MC13b) and one affected case (MC13) in family B because of the error in information transfer (the affected person was switched from MC13a to MC13b), theoretically resulting in one mutant allele among four existing alleles. However, we still detected a pathological variant (c.124delT), which was present at an allele frequency of 36.47% in our sequence reads. This is consistent with recent reports that WES can detect mosaic pathogenic mutations present at allele frequencies as low as 3.6% to 8% [21-24]. WES has been proven to be useful in clinical diagnosis and personalized disease-risk profiling [10]. Several groups applied WES to successfully identify de novo pathogenic mutations in sporadic patients, supporting its utility [25-27]. WES is particularly useful for small pedigrees, in which linkage mapping is difficult, for cases with previously unrecognized or atypical phenotypes, and for disorders with high genetic heterogeneity [28,29]. Because congenital cataract shows wide phenotypic variability and genetic heterogeneity, WES is appropriate to reach a correct genetic diagnosis. In fact, we performed WES in three families showing congenital cataract and identified pathogenic mutations in two as described here, supporting that WES is quite powerful for dissecting the genetic basis of congenital cataract. Because the cost of WES is now falling, it is likely to be provided as a clinical service in the very near future and will provide useful information for genetic counseling and family planning in congenital cataract. In conclusion, WES successfully identified pathogenic mutations in two Korean families with congenital cataract, clearly demonstrating the efficiency and diagnostic utility of this technique in congenital cataract.

## Appendix 1.

Whole-exome sequencing performance. To access the data, click or select the words “Appendix 1.”

## Appendix 2.

Candidate genes for congenital cataract. To access the data, click or select the words “Appendix 2.”
